# Reply to “Spinal Cord Atrophy Is a Preclinical Marker of Progressive MS”

**DOI:** 10.1002/ana.26340

**Published:** 2022-03-26

**Authors:** Antje Bischof, Nico Papinutto, Anisha Keshavan, Anand Rajesh, Gina Kirkish, Xinheng Zhang, Jacob M. Mallott, Carlo Asteggiano, Simone Sacco, Tristan J. Gundel, Chao Zhao, William A. Stern, Eduardo Caverzasi, Yifan Zhou, Refujia Gomez, Nicholas R. Ragan, Adam Santaniello, Alyssa H. Zhu, Jeremy Juwono, Carolyn J. Bevan, Riley M. Bove, Elizabeth Crabtree‐Hartman, Jeffrey M. Gelfand, Douglas S. Goodin, Jennifer S. Graves, Ari J. Green, Jorge R. Oksenberg, Emmanuelle Waubant, Michael R. Wilson, Scott S. Zamvil, University of California, San Francisco MS‐EPIC Team, Bruce A. Cree, Stephen L. Hauser, Roland G. Henry

**Affiliations:** ^1^ Weill Institute for Neurosciences Department of Neurology, University of California, San Francisco San Francisco CA; ^2^ Department of Neurology with Institute for Translational Neurology University Hospital Münster Münster Germany

Zeydan et al underline the contribution of their work to the current knowledge of spinal cord atrophy and its usefulness as a biomarker of progressive multiple sclerosis (MS). After the early publications by Kidd and colleagues^1^, ^2^ suggesting a strong correlation between disability and spinal cord atrophy at various cord levels in progressive MS, Filippi et al reported that cervical cord atrophy appears to be absent in benign MS but severe in secondary progressive MS (SPMS).^3^ Many subsequent studies confirmed and extended these findings, corroborating the role of spinal cord atrophy as a promising magnetic resonance imaging biomarker of disability and progressive disease in MS.^4^, ^5^, ^6^, ^7^, ^8^, ^9^, ^10^ Associations of cord atrophy with long‐term disability pointed to its potential as an outcome measure in MS.^11^ Later, evidence from longitudinally designed studies indicated that spinal cord atrophy rates were linked to disability worsening whereas baseline total cord area was not.^12^ Eventually, a strong predictive value of spinal cord atrophy rates for disability worsening was shown in a mixed relapsing–remitting MS (RRMS)/SPMS cohort over 6 years by introduction of a more sophisticated statistical approach using survival analyses.^13^

In their cross‐sectional study, Zeydan et al assessed total cord area shortly after SPMS conversion at the C2, C7, and C2–C7 levels from T2‐weighted clinical protocols. In contrast to our study,^14^ they did not find differences between RRMS and SPMS patients at the high cervical cord level.^15^ However, cord area at the C7 level discriminated between the two groups, suggesting that caudal levels were more sensitive to disability than those in the high cervical cord, an analysis not undertaken in our study. As mentioned by the authors, this finding was confirmed and extended by Rocca et al,^16^ who more comprehensively assessed the entire cervical cord at each vertebral level.

However, as the authors state in their letter, they could only hypothesize on the occurrence of spinal cord atrophy during the preceding relapsing phase due to the cross‐sectional nature of their study. By contrast, our study is the first to longitudinally assess patients during the preceding relapsing phase before they converted to SPMS, some as early as 12 years before conversion. This allowed us to evaluate the predictive value of brain and C1A (cervical cord area at the C1 vertebral level) measures for silent progression and SPMS conversion. Of note, this long observational period exceeds that of diagnostic uncertainty due to an insidious clinical worsening, which could have confounded the results by Zeydan et al.^17^ Our finding that C1A rates are predictive of SPMS is clearly novel. The result also calls into question the concept of a dichotomy between RRMS and SPMS, and suggests that a redefinition of SPMS based on more sensitive measures including cord atrophy rates might be considered. Finally, we recently reported that spinal cord gray matter area might be an even more sensitive indicator of future outcome in MS as the total cord area can be larger in patients than controls during early disease and in those patients who do not progress clinically.^18^, ^19^

## Potential Conflicts of Interest

The following companies that have had financial relationships with authors manufacture disease‐modifying drugs for MS that were mentioned in this study: Bayer Schering, Biogen, EMD Serono, Genentech, Genzyme, F. Hoffmann‐La Roche, Mylan Pharmaceuticals, Novartis, Sanofi, Teva Pharmaceuticals. R.M.B. has received personal compensation for medical legal consulting and for consulting or serving on the advisory boards of F. Hoffmann‐La Roche, Sanofi‐Genzyme, and Novartis. J.M.G. reports consulting fees from Biogen and research support from Genentech. D.S.G. has received research support and given lectures on MS and its treatment that have been sponsored by Biogen Idec, Bayer Schering, Novartis, EMD Serono, Genzyme, and Teva Pharmaceuticals. J.S.G. reports personal fees from Novartis and Genentech and grants from Biogen, and EMD Serono. A.J.G. reports personal fees from Mylan Pharmaceuticals, grants from Novartis, and payments for serving on committees from Biogen and Novartis. E.W. has received research support from Novartis and Roche. M.R.W. receives research support from Roche/Genentech. S.S.Z. has served as a consultant and received honoraria from Biogen Idec, EMD Serono, Genzyme, Novartis, Roche/Genentech, and Teva Pharmaceuticals, and has served on data safety monitoring boards for Teva. B.A.C. has received personal compensation for consulting from Biogen, EMD Serono, and Novartis. R.G.H. received grants from Hoffmann La Roche, and consultancy honoraria from Roche/Genentech, Sanofi/Genzyme, and Novartis. S.L.H. has received travel reimbursement and writing assistance from F. Hoffman‐La Roche and Novartis Pharma. The other authors have nothing to report.
